# Leveraging Blockchain Technology for Informed Consent Process and Patient Engagement in a Clinical Trial Pilot

**DOI:** 10.30953/bhty.v4.182

**Published:** 2021-10-15

**Authors:** Baldwin C. Mak, Bryan T. Addeman, Jia Chen, Kim A. Papp, Melinda J. Gooderham, Lyn C. Guenther, Yi Liu, Uli C. Broedl, Marianne E. Logger

**Affiliations:** 1 Department of Clinical Operations, Boehringer Ingelheim Canada Ltd./Ltée, Burlington, ON, Canada; 2 International Business Machines (IBM), Markham, ON, Canada; 3 International Business Machines (IBM), Yorktown Heights, NY, USA; 4 K. Papp Clinical Research and Probity Medical Research Inc., Waterloo, ON, Canada; 5 SKiN Centre for Dermatology, Peterborough, ON, Canada; 6 The Guenther Dermatology Research Centre, London, ON, Canada; 7 Boehringer Ingelheim Pharmaceuticals, Ridgefield, CT, USA; 8 Global Clinical Development & Operations, Boehringer Ingelheim Pharma GmbH & Co. KG, Ingelheim, Germany

**Keywords:** blockchain, clinical trials, healthcare and medical research, informed consent, Hyperledger Fabric

## Abstract

**Objective:**

Despite the implementation of quality assurance procedures, current clinical trial management processes are time-consuming, costly, and often susceptible to error. This can result in limited trust, transparency, and process inefficiencies, without true patient empowerment. The objective of this study was to determine whether blockchain technology could enforce trust, transparency, and patient empowerment in the clinical trial data management process, while reducing trial cost.

**Design:**

In this proof of concept pilot, we deployed a Hyperledger Fabric-based blockchain system in an active clinical trial setting to assess the impact of blockchain technology on mean monitoring visit time and cost, non-compliances, and user experience. Using a parallel study design, we compared differences between blockchain technology and standard methodology.

**Results:**

A total of 12 trial participants, seven study coordinators and three clinical research associates across five sites participated in the pilot. Blockchain technology significantly reduces total mean monitoring visit time and cost versus standard trial management (475 to 7 min; *P* = 0.001; €722 to €10; *P* = 0.001 per participant/visit, respectively), while enhancing patient trust, transparency, and empowerment in 91, 82 and 63% of the patients, respectively. No difference in non-compliances as a marker of trial quality was detected.

**Conclusion:**

Blockchain technology holds promise to improve patient-centricity and to reduce trial cost compared to conventional clinical trial management. The ability of this technology to improve trial quality warrants further investigation.

The COVID-19 pandemic has impelled significant transformations in the clinical trial development process that have led to improved efficiency in generating high-quality data (1). Efforts that primarily aim to enhance patient recruitment, comfort, and retention are: adopting digital patient engagement, trial virtualisation, and site support tools. The introduction of telemedicine, mobile, or local healthcare providers coupled with novel information technology solutions holds promise for improving patient engagement and trial performance. However, rigorous management of clinical trial data remains a fundamental concern. The current data management process requires that data be captured and stored among different centralised databases. This process necessitates data entry duplication across multiple platforms, requiring cross-checking and manual source data verification (SDV) to ensure data quality. In addition to being time-consuming, these processes are also susceptible to error and unauthorised access to personal healthcare information.

In an annual Health Canada Good Clinical Practice (GCP) inspection summary report, the two most common observations noted by GCP inspectors were related to the lack of systems and procedures to ensure data quality (40%) as well as errors/incomplete records (30%) (2). Issues with records were also noted as a common observation for the Food and Drug Administration (FDA), European Medicines Agency (EMA), and Medicines and Healthcare products Regulatory Agency (MHRA), in their retrospective 2016 inspection summary reports (3, 4). Quality assurance procedures, which typically include traditional methods such as intensive on-site monitoring with 100% SDV at 4- to 8–week intervals, are put into place to prevent these findings (5, 6). These quality assurance measures also provide confidence among the many different stakeholders in a clinical trial (i.e. participants, principal investigators, clinical sites, sponsors, and regulators), and are necessary to maintain the trust and transparency in these relationships.

The relationships among trial participants, clinical sites, ethics committees, regulators, and trial sponsors are crucial for the success of a clinical trial. In particular, the rights, safety, and well-being of trial subjects are the most important considerations (7, 8). Informed consent is the cornerstone of the conduct of any ethical human subject research – it informs participants about the trial objective, trial flow, benefits, and risks, which in turn empowers them to enrol in a study voluntarily. It is imperative, as participants’ trust and comfort can impact many aspects of a trial, such as recruitment, protocol adherence, and study completion. Trial participants convey their confidence and willingness to participate in a trial by providing informed consent.

The informed consent process, which is predominantly paper-based, is susceptible to errors as consent forms are often long, convoluted, and complex in nature. Additionally, poor communication between parties can lead to a lack of informed consent because of a failure to perform reconsent procedures that may be required prior to the implementation of protocol changes. Between 2015 and 2016, informed consent observations ranged from approximately 2–11% of the annual inspection findings among Health Canada, EMA, MHRA, and the FDA (2–4, 9). Failure to adhere to a participant’s informed consent, and any changes to this status, violates International Conference on Harmonisation Good Clinical Practice (ICH-GCP) standards, and may put the trial participants’ rights and safety at risk.

Addressing critical challenges of trust, transparency, patient empowerment, and patient safety requires a new clinical trial and data management model. This model should complement future technological transformations in healthcare environments and systems, in which we envision fully digital platforms, remote site monitoring, integration of wearable devices for data collection, and automated data-curating mechanisms. We hypothesise that an emerging technology, known as blockchain technology, may support a future clinical trial model based on the intrinsic benefits this technology promises.

Blockchain technology is designed as a distributed, decentralised, and immutable digital ledger shared within a network of stakeholders (10). Members of a blockchain network have access to a copy of the ledger – a shared single ‘source of truth’. Consensus algorithms validate new transactions and agreements, which are then added into the ledger and chained to previous entries in ‘blocks’ (10, 11). Consensus is the process by which a network of blockchain nodes provides a guaranteed ordering of transactions and validates the block of transactions. It confirms the correctness of all transactions in a proposed block, according to endorsement and consensus policies. Typical consensus types include: Proof of Work, Proof of Stake, Proof of Elapsed Time, or Redundant Byzantine Fault Tolerance (RBFT). Each copy of the ledger is updated with each new block and becomes visible to all network members. Altering an entry in the ledger is almost impossible as modification requires authorisation from a majority of stakeholders and changing all previous entries (12). This technology creates ownership and gives each network member a stake in knowing and deciding what happens with their own data. Thus, blockchain inherently has the potential to enhance trust, transparency, and empowerment among members who share the ledger. Smart contracts (i.e. code stored on blockchain that automatically executes under predetermined conditions) allow for process automation in a trusted environment without third-party intervention and, therefore, may lead to improved process quality at reduced cost (12–14).

The concept of blockchain technology in clinical trials has previously been explored (15). However, to our knowledge, it has not been evaluated in an active clinical trial setting. Thus, we have designed a blockchain pilot as a sub-study to a clinical trial to test the hypothesis that blockchain technology creates trust, transparency, and patient empowerment, in addition to improving trial quality and patient safety at a reduced cost compared to the current standard for clinical trial management.

## Methods

### Blockchain pilot design

**Fig. 1:**
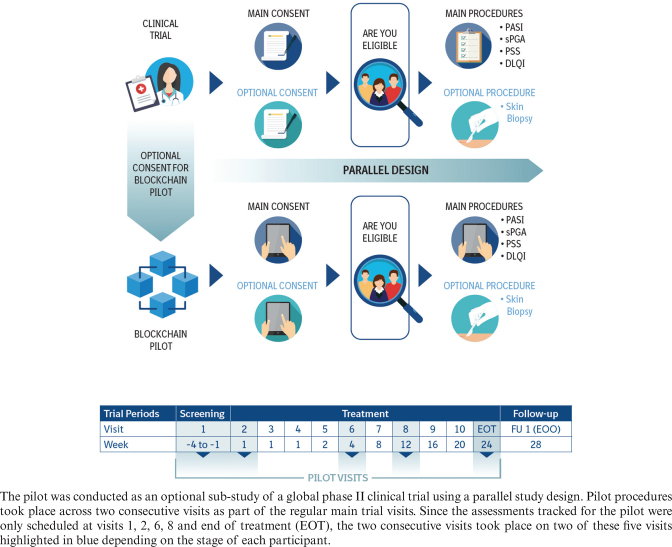
Blockchain pilot study design.

The blockchain pilot was designed as a proof-of-concept sub-study of a global phase II clinical trial (NCT03635099, referred to as the ‘main trial’) using a parallel study design (see Fig. 1) to assess the value proposition of blockchain technology versus conventional trial management. The pilot, which was approved by Health Canada, was conducted between March 2019 and November 2019, and was limited to Canadian sites and trial participants. Participants who signed the informed consent of the main trial could choose to enrol in the pilot by signing a separate optional consent form.

Since the pilot was introduced after the main trial had already started, participants could either join at the time of recruitment for the main trial or, for those already enrolled in the main trial, at their next planned visit. Each pilot participant followed the visit schedule and procedures of the main trial as outlined in the clinical trial protocol. Procedures for the pilot took place across two consecutive planned visits as part of their regular visits for the main trial (two of the five blue highlighted visits in Fig. 1). At the first pilot visit, participants acknowledged their prior consent for the main trial and, if applicable, an optional skin biopsy through a ‘patient’ portal on a tablet device. The study coordinator counter acknowledged the consent status through a ‘site’ portal. At the next visit, the consensus of the status for the consents was used as a directive to pilot participants and site coordinators for the completion of five procedures (i.e. Psoriasis Area and Severity Index [PASI], static Physician’s Global Assessment [sPGA], Psoriasis Symptom Scale [PSS], Dermatology Life Quality Index [DLQI], and skin biopsy). These procedures are standard dermatological assessments. Pilot participants and study coordinators were required to confirm the completion of these procedures through the portal once they were done in the main trial.

### Technical details of blockchain

**Fig. 2:**
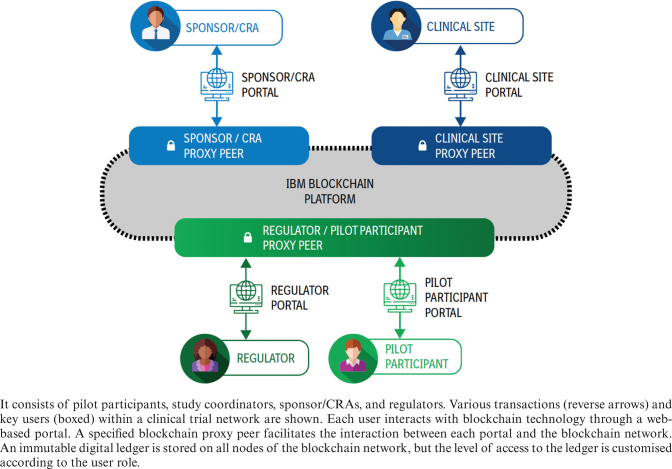
Overview of the private, permissioned IBM blockchain platform.

The blockchain system was implemented using the open-source Hyperledger Fabric v1.4 project from the Linux Foundation, and was deployed using the IBM blockchain platform (Fig. 2). The platform was enterprise-grade and built on a private, permissioned network. A set of blockchain consent services were leveraged to enable pilot participants to grant or withdraw informed consent, to reconsent during the trial process, and to secure exchange of clinical trial status data. The platform was designed for the following users: pilot participants, study coordinators, and sponsor staff – which included both clinical trial managers and clinical research associates (CRAs). All users were known and identified by cryptographic keys. Information that the users were able to view and update was customised according to their role. Each user role interacted with the blockchain network via a web application or portal. The informed consent status recorded on blockchain was leveraged as a directive for the completion of the tracked procedures. This allowed all users to see the consent status, what procedures the pilot participant agreed to, what should happen at the next visit, and whether the procedure was completed through the user portal.

The pilot participant portal permitted participants to confirm the status of their consent/reconsent in the main trial, their optional consent, their withdrawal, the completion of selected trial procedures, and to be alerted to participant versus study coordinator entry mismatches in near real–time. Clinical site portals permitted study coordinators to confirm participant informed consent and withdrawal status, confirm participant eligibility, monitor participant informed consent and trial status per trial participant (and in aggregate), and be alerted to participant versus site entry mismatches in near real–time. Sponsor portals permitted sponsors to create and populate the details of the trial (e.g. update informed consent versions), monitor informed consent and clinical trial status per site (and per participant), and monitor participants versus site entry mismatches in near real–time. A regulator portal was designed to model outputs needed by a regulatory authority. This portal provided near real–time, read-only access to participant status and conduct in trials across multiple sponsors.

Each portal interacted with the blockchain network (via a specified blockchain peer) where an immutable record of the pilot participant consent directive and trial progress were stored. The peers applied the permissions defined by the custom rules of this private network such that a retrieval or creation/update of a record could only be possible for an authenticated permissioned user of the appropriate user role. Because of the nature of the technology, an immutable audit log of all interactions was available within the blockchain network.

### Pilot endpoints

Endpoints of the blockchain pilot comprised clinical trial monitoring time and costs, number of non-compliances (as a proxy for trial quality), as well as user-reported outcomes of trust, transparency, and sense of empowerment.

### Data collection and analysis

Monitoring for the main trial was conducted on-site, while monitoring for the blockchain pilot was conducted remotely using the sponsor portal. Total monitoring visit time comprised time spent on review of informed consent status and completion of the tracked procedures, on query follow-up, and on other monitoring visit activities, which included travel time and waiting time as part of on-site visits, and systems login and visit preparation time as part of either on-site or remote visits. Monitoring visit time was self-reported by the CRA, and was only compared for pilot participants who took part in both the main trial and pilot study. Pilot participants served as their own control. Estimated cost for monitoring was calculated using monitoring visit time and an industry standard CRA hourly rate. Additional expenses incurred for monitoring visits, such as pass-through costs (i.e. hotel, transportation, and meals), were not included in the cost estimates.

Events of non-compliance pertaining to completion of informed consent and conduct of tracked procedures were captured in the pilot and main trial without unblinding of treatment allocation of trial participants. The number of non-compliances in the pilot and main trial was assessed, with pilot participants serving as their own control. As participation in the blockchain pilot may have influenced compliance of the pilot participants in the main trial (and vice versa), the total number of non-compliances related to informed consent and tracker procedures for Canadian main trial participants who joined the blockchain pilot was also assessed.

Trust, transparency, and sense of empowerment of pilot participants, study coordinators, and CRAs were assessed using surveys that were developed based on TransCelerate Patient Experience Initiative guidance (16). A 5-point Likert scale system was used for the responses in the surveys. Responses were based on the level of agreement to a statement: 1) strongly disagree; 2) disagree; 3) neither agree nor disagree; 4) agree; and 5) strongly agree. In the analysis of the user survey results, data were combined resulting in three levels of agreement: 1) disagree, 2) undecided, and 3) agree. Users completed the surveys at the end of the pilot trial.

### Statistical analysis

Baseline demographics and clinical characteristics were summarised for pilot participants, using SAS^®^, version 9.4 (SAS Institute). All analyses of the endpoints of the pilot study were exploratory. The data for monitoring time and costs are presented as the mean ± standard deviation on a log scale. Data were analysed by a two-sided t-test for two-group comparisons, using GraphPad Prism software. A *P* < 0.05 was statistically significant.

## Results

### Pilot disposition and demographics

There were eight Canadian sites with 36 participants in the global phase II psoriasis main trial. Twelve (33.3%) of the 36 participants from five (62.5%) of the eight sites voluntarily chose to enrol in the blockchain pilot. Nine (75%) of the 12 pilot participants enrolled with the original main informed consent form and were reconsented with a revised main informed consent while the pilot was conducted, whereas three (25%) of the 12 participants directly enrolled with a revised main consent (Table 1). Most of the pilot participants (92.7%) were Caucasian, with a mean age of 52.7 years. The pilot patient group had a mean PASI score of 15.4, consistent with moderate to severe psoriasis (Table 2).

**Table 1:** Blockchain pilot site profile and participant groups

Main Trial	Total Sites	8
	Total Participants	36
Pilot	Trial Sites	5
Sub-study	Total Pilot Participants	12
	Enrolled with revised main consent Enrolled and reconsented with revised main consent	3
		9
	Study Coordinators	7
	Clinical Reseach Associates	3

**Table 2:** Baseline demographic and clinical characteristics of pilot participants

Number of pilot participants (N, %)	12 (100.0)
Race (N, %)	11 (91.7)
White	0 (0)
African American or Black Asian	1 (8.3)
Age, mean (Years, SD)	52.7 (16.6)
Mean Body Surface Area (BSA) Affected With Plaque Psoriasis (%, SD)	16.2 (6.7)
Mean PASI Score (N, SD)	15.4 (2.6)
sPGA Score (N, %)	
Moderate	8 (66.7)
Severe	4 (33.3)
Mean PSS Score (N, SD)	8.0 (4.0)
Mean DLQI Score (N, SD)	10.8 (6.8)
Trial participants with at least one concomitant diagnosis (N, %)	11 (91.7)
Trial participants who took non-topical psoriasis (N, %)	6 (50.0)
Trial participants with at least one on-treatment concomitant medication (N, %)	9 (75.0)

SD=standard deviation; PASI=Psoriasis Area and Severity Index; sPGA= Static Physician’s Global Assessment; PSS=Psoriasis Symptom Scale; DLQI= Dermatology Life Quality Index.

### Monitoring visit time and costs

**Fig. 3:**
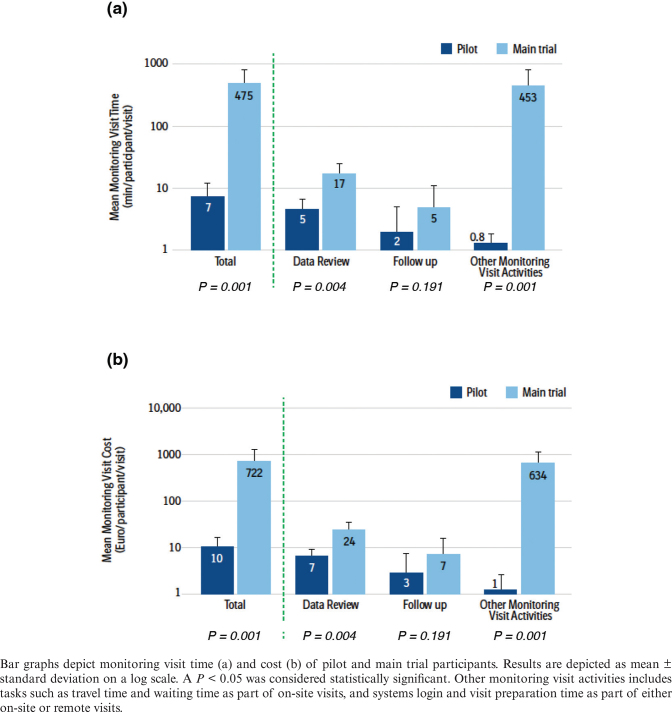
Mean monitoring visit time and cost.

The total mean monitoring visit time for each participant per visit was significantly reduced from 475 min in the main trial to 7 min in the blockchain pilot (*P* = 0.001). This decrease was largely attributed to the significant reduction in time spent on other monitoring visit activities (i.e. travel time, waiting time as part of on-site visits, and systems login and visit preparation time as part of either on-site or remote visits), from 453 to 0.8 min (*P* = 0.001). Time spent for monitoring tasks was also reduced (data review [from 17 to 5 min; *P* = 0.0004]; follow-up [from 5 to 2 min; *P* = 0.191]) (Fig. 3a). As a result of the reduction in monitoring visit time, a significant reduction in total mean cost for monitoring visit activities was observed (from €722 to €10; *P* = 0.001). This result was primarily driven by reduced costs for other monitoring visit activities (from €634 to €1; *P* = 0.001) and for data review (from €24 to €7; *P* = 0.0004) (Fig. 3b).

### Non-compliance

There was one incident of non-compliance in the pilot group and no incident of non-compliance in the same patients participating in the main trial. Due to a technical issue with the patient portal, one pilot participant could not confirm the successful completion of reconsent, resulting in a mismatch with the site entry as documented on-chain. Overall, there was only one incident of non-compliance in the main trial, including non-blockchain trial participants in Canada, precluding any assessment of the hypothetical value proposition of blockchain technology regarding study compliance and quality in our pilot.

## User survey results

### Pilot participants

**Fig. 4:**
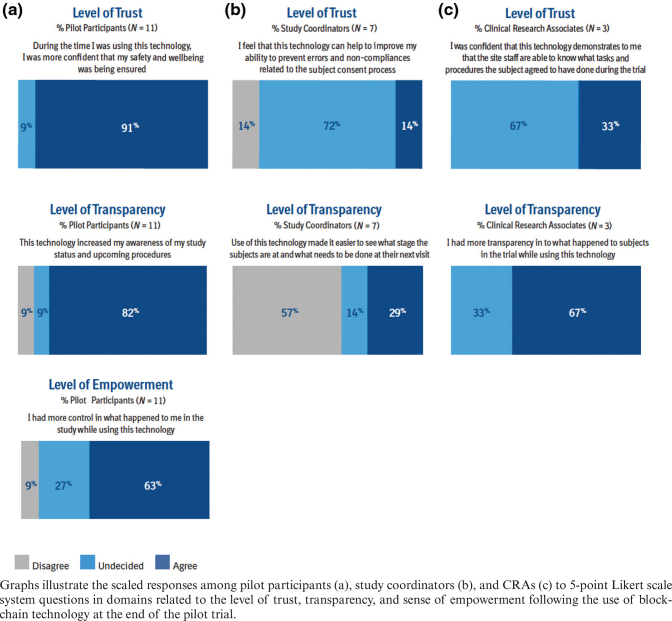
User survey results on use of blockchain technology in a clinical trial setting.

Eleven of the 12 pilot participants completed a user survey to determine the impact of blockchain technology on trust, transparency, and sense of empowerment (Fig. 4a). The majority (91%) of survey respondents indicated that blockchain technology increased their confidence that their safety and well-being were being ensured; 82% of respondents felt that blockchain technology increased their awareness regarding their trial status and upcoming procedures; 63% of respondents indicated that they had more control over what happened to them in the trial while using blockchain technology.

### Study coordinators

Seven study coordinators completed a user survey to determine the impact of blockchain technology on trust and transparency in their role as site representatives (Fig. 4b). The majority (72%) of coordinators were undecided when asked if they felt that the technology improved their ability to prevent errors and non-compliances related to the pilot participant consent process. Among the remaining respondents, there was an equal divide among those who agreed (14%) or disagreed (14%). Fifty-seven percent (57%) of coordinators disagreed that blockchain technology made it easier for them to see what stage the pilot participants were at and what needed to be done at their next visit, while 29% agreed that the technology improved transparency, and 14% remained undecided.

### Clinical research associates

Three CRAs completed a user survey to determine the impact of blockchain technology on trust and transparency as sponsor representatives (Fig. 4c). When asked about their confidence in blockchain’s ability to demonstrate that the study coordinator was aware of what subjects agreed to during the trial, 67% of CRAs were undecided, while 33% agreed. The majority of CRAs (67%) agreed that they had more transparency about what happened to participants in the trial when using the technology, while 33% of CRAs were undecided.

## Discussion

In this pilot, we aimed to assess the value proposition of blockchain technology in an active clinical trial setting. Our data suggest that blockchain technology reduces monitoring visit time and cost while improving patient trust and sense of empowerment compared to conventional clinical trial management.

The cost of clinical trials continues to rise annually primarily driven by intricate trial design and operational setup choices. As trial monitoring to prevent and remedy non-compliance is estimated to contribute 25–30% of the overall clinical trial costs, process automation via smart contracts in a trusted environment using blockchain technology appeared to be a promising approach to improve trial quality and reduce monitoring efforts (17). While the resulting reduction in trial costs supports our hypothesis, our study could not answer whether blockchain technology truly enhanced trial quality and process compliance because of the small number of non-compliances observed given the limited number of pilot participants and our efforts to run high-quality clinical trials.

We also hypothesised that blockchain technology could enhance patient-centricity in clinical trials by providing transparency, safety, trust, and empowerment. The pilot provided valuable insight into the attitudes and preferences of patients, study coordinators, and CRAs through the assessment of qualitative data. The majority of patients appreciated the value proposition of blockchain technology; however, the responses from study coordinators and CRAs were mixed. This result may be due, in part, to a change in the operating model and the parallel execution and timing of the pilot within the main trial. Adjustments in the design of the user portal and how trial information is displayed are other considerations that could improve transparency for site coordinators and CRAs.

Our pilot study had several strengths and limitations. To our knowledge, this is the first assessment of blockchain technology in an active clinical trial setting. While several proof-of-concept studies using artificial or existing patient data have suggested that blockchain technology may improve trust, transparency, and auditability of clinical trials in addition to patient empowerment, our parallel study design allowed us to prospectively compare the value proposition of blockchain technology versus conventional trial management (10, 18, 19). Of note, since blockchain technology is not currently accepted by health authorities to support routine clinical trials, the cost savings reported here remain elusive. The low number of pilot participants may have impacted our ability to detect any differences in non-compliances and trial quality. However, it did offer us the unique opportunity to manually verify all blockchain-based transactions against conventional process steps, strengthening the validity of our conclusions.

In summary, the use of blockchain technology in clinical trials holds the promise of improving trust, transparency, auditability, patient empowerment, and clinical trial costs. The ability of blockchain technology to improve trial quality and patient safety remains unanswered and warrants further investigation. Of note, despite the promising value proposition of blockchain technology in clinical trials, broad adoption will require the industry to overcome technological barriers, including scalability and interoperability across different blockchain solutions, as well as non-technological barriers such as the cross-functional development of blockchain knowledge, skills, and regulatory frameworks.
